# Programmed Cell Death-1 Deficiency Exacerbates T Cell Activation and Atherogenesis despite Expansion of Regulatory T Cells in Atherosclerosis-Prone Mice

**DOI:** 10.1371/journal.pone.0093280

**Published:** 2014-04-01

**Authors:** Clément Cochain, Sweena M. Chaudhari, Miriam Koch, Heinz Wiendl, Hans-Henning Eckstein, Alma Zernecke

**Affiliations:** 1 Institute of Clinical Biochemistry and Pathobiochemistry, University Hospital Würzburg, Würzburg, Germany; 2 Department of Vascular Surgery, Klinikum rechts der Isar, Technical University Munich, Munich, Germany; 3 Department of Neurology, University of Münster, Münster, Germany; 4 DZHK (German Centre for Cardiovascular Research), partner site Munich Heart Alliance, Munich, Germany; Wayne State University, United States of America

## Abstract

T cell activation represents a double-edged sword in atherogenesis, as it promotes both pro-inflammatory T cell activation and atheroprotective Foxp3^+^ regulatory T cell (Treg) responses. Here, we investigated the role of the co-inhibitory receptor programmed cell death-1 (PD-1) in T cell activation and CD4^+^ T cell polarization towards pro-atherogenic or atheroprotective responses in mice. Mice deficient for both low density lipoprotein receptor and PD-1 (*Ldlr^−/−^Pd1^−/−^*) displayed striking increases in systemic CD4^+^ and CD8^+^ T cell activation after 9 weeks of high fat diet feeding, associated with an expansion of both pro-atherogenic IFNγ-secreting T helper 1 cells and atheroprotective Foxp3^+^ Tregs. Importantly, PD-1 deficiency did not affect Treg suppressive function *in vitro*. Notably, PD-1 deficiency exacerbated atherosclerotic lesion growth and entailed a massive infiltration of T cells in atherosclerotic lesions. In addition, aggravated hypercholesterolemia was observed in *Ldlr^−/−^Pd1^−/−^* mice. In conclusion, we here demonstrate that although disruption of PD-1 signaling enhances both pro- and anti-atherogenic T cell responses in *Ldlr^−/−^* mice, pro-inflammatory T cell activation prevails and enhances dyslipidemia, vascular inflammation and atherosclerosis.

## Introduction

Atherosclerosis, the major cause of myocardial infarction and stroke, is a chronic inflammatory disease of the vascular wall characterized by accumulation of lipid-laden macrophages in the vascular intima [1]. It is now well accepted that local and systemic adaptive immune responses are also a major determinant of atherogenesis, and several CD4^+^ T helper (Th) cell subsets have been shown to promote or mitigate atherosclerotic lesion progression. While interferon (IFN)-γ secreting Th1 cells promote atherogenesis [2], Foxp3^+^ regulatory T cells (Treg) inhibit lesion formation through attenuation of adaptive and innate immune responses [3], [4]. The role of other polarized T cell subsets is less clear. The role of Th2 cells in atherogenesis remains to be conclusively defined, as this subset was proposed to limit atherogenesis, in part through IL-13 secretion [3], whereas other Th2-cytokines, in particular IL-4, might be pro-atherogenic [4], [2]. Likewise, IL-17-secreting Th17 cells have been proposed to both promote and limit atherogenesis [4].

T helper cell activation during adaptive immune response, including hypercholesterolemia-associated immunity, requires three distinct signals, namely TCR stimulation, co-stimulatory or -inhibitory signals, cytokine mediated potentiation or attenuation of T cell responses [5]. It has been previously shown that co-stimulatory molecules of the B7/CD28 and tumor necrosis factor (TNF)/TNF receptor family are instrumental in T cell activation during atherosclerosis [6]. Mice deficient for both B7-1 and B7-2 in atherosclerosis prone Low density lipoprotein receptor-deficient (*Ldlr^−/−^*) mice demonstrated impaired T cell activation, and in turn diminished atherosclerosis [7]. Antibody mediated targeting of CD40L or CD40 deficiency in *Ldlr^−/−^* or apolipoprotein E-deficient (*Apoe^−/−^*) mice, respectively, similarly decreased vascular inflammation and atherosclerosis [8], [9], [10].

However, also atheroprotective Treg functions depend on similar co-stimulatory signals [6]. *Ldlr^−/−^* mice irradiated and reconstituted with bone marrow cells deficient in B7-1 and B7-2 or CD28 displayed impaired Treg homeostasis and increased atherosclerosis [8]. Although discrepancies between studies using B7-1/2-deficient mice on a hypercholesterolemic background and studies employing bone marrow transplantation are unexplained, it appears that bone marrow chimerism preferentially affects Treg activation, leading to a pro-atherogenic phenotype despite reduced effector T cell activation. In line, chimeric *Ldlr^−/−^* mice carrying bone marrow deficient in Inducible Co-stimularoy Molecule (ICOS) displayed increased atherosclerosis, presumably associated with decreased Treg levels, while lesion formation was not affected in *Icos^−/−^Ldlr^−/−^* double knockout mice [6], [9]. Recently, *Ldlr^−/−^* mice irradiated and reconstituted with *CD11c^cre^MyD88^flox^* bone marrow, which renders dendritic cells insensitive to Toll-like receptor-induced maturation, were shown to develop increased atherosclerosis despite strikingly decreased T cell activation, and it was proposed that the failure to mount adequate Treg responses was able to overcome anti-atherogenic effects of depressed effector T cell activation [9]. The overall outcome of co-stimulation thus seems to represent a fine-tuned balance between activation of pro-atherogenic effector T cell and immunomodulatory Treg responses.

The co-inhibitory receptor Programmed Cell Death-1 (PD-1) belongs to the CD28 family and is essential in T cell tolerance [10], [11]. PD-1 is expressed by T cells and binds to PD-L1 and PD-L2, widely expressed by a number of cells at different levels [10], [11]. Of note, both PD-L1 and PD-L2 can be expressed on activated DCs [10]-[12]. Although PD-1 and its ligands have been described as an essential anti-atherogenic pathway [10], [11], the role of PD-1 in Treg homeostasis during hypercholesterolemia is unknown. Employing *Pd1^−^* ^/*−*^ *Ldlr^−/−^* mice, we here show that deficiency in PD-1 increased T cell activation during atherogenesis despite a strong increase in systemic Foxp3^+^ regulatory T cells, and accelerated atherogenesis associated with a massive T cell infiltration in lesions.

## Material and Methods

### Mice and diet

*Pd1^−/−^* mice were crossed with *Ldlr^−/−^* mice (both on a C57BL/6J background, Jackson Laboratory), and housed under pathogen free conditions. At 8 weeks of age, male *Ldlr^−/−^Pd1^−/−^*and *Ldlr^−/−^*mice were placed on an atherogenic diet (15% milk fat, 1.25% cholesterol, Altromin, Germany) for 9 weeks. All animal experiments were approved by local authorities (Regierung von Unterfranken, Würzburg, Germany, Akt.-Z. 55.2-2531.01-37/09) to comply with German animal protection law.

### Flow cytometry

Blood counts were analyzed using an automated hematology analyzer (KX-21N, Sysmex, Germany). For FACS analyses, tissues were passed through a 70 μm filter (BD Biosciences, Germany) to obtain single-cell suspensions. Whole blood was combined with a red blood cell lysis buffer (155 mM NH_4_Cl, 10 mM KHCO_3_, 0.1 mM EDTA) to allow the isolation of leukocytes. Whole aortae and aortic roots were excised, flushed with PBS, and enzymatically dissociated using Liberase Blendzyme TL (Roche, Germany) solution for 30 minutes at 37°C. The resulting single cell suspensions were resuspended in Hanks Buffered Saline Solution (HBSS), enumerated using a Neubauer counting chamber. Cells were stained for 30 minutes on ice using combinations of specific antibodies from BD biosciences (CD45, clone 30-F11; CD3, clone 500A2; CD8a, clone 53-6.7; IFNγ, clone XMG1.2) and eBioscience (TCRβ, clone H57-597; CD44, clone IM7; CD62L, clone MEL-14; CD4, clone RM4-5; Foxp3, clone FJK-16s; CD25, clone PC61.5; IL-17a, clone eBio17B7). Intracellular labelling of IL17A and IFNγ was performed using the BD Cytofix/Cytoperm Kit (BD Biosciences). Intracellular labeling of Foxp3 was performed using the Foxp3 Staining Buffer Set (eBioscience) according to the manufacturer's instructions. Probes were analyzed using a FACSCanto II (Becton Dickson, USA) and FlowJo 7.6 software (Treestar Inc., USA).

### *In vitro* Treg suppression assay

Spleen and peripheral (inguinal) lymph node regulatory T cells from *Ldlr^−/−^* and *Ldlr^−/−^Pd1^−/−^* mice and naïve T cells from CD45.1 mice were enriched using magnetic microbeads (CD4^+^CD25^+^ Regulatory T cell isolation kit, CD4^+^CD62L^+^ naïve T cell isolation kit), according to the manufacturer's instruction (Miltenyi Biotec, Bergisch Gladbach, Germany). Isolated naïve T cells were stained with carboxyfluorescein succinimidyl ester (CFSE) and mixed with regulatory T cells at ratio of 2∶1. Cells were stimulated with 2.5 μg/ml rat anti-mouse CD3 (clone 145-2C11) and 2.5 μg/ml rat anti-mouse CD28 (clone 37.51). After 48 hours, cells were washed and stained with anti-CD4 (clone RM4-5) and anti-CD45.1 (clone A20) antibodies. Proliferation of CD45.1 naïve T cell was analyzed by CFSE dye dilution.

### Assessment of atherosclerosis and immunofluorescence

Arteries were perfusion-fixed *in situ* with phosphate buffered saline (PBS) followed by 4% paraformaldehyde in PBS (PFA; Sigma Aldrich, USA). The heart and whole aorta were removed and carefully cleaned of extraneous fat before being post-fixed in 4% PFA. The heart was embedded into paraffin and cut into 5-μm transverse sections. Aortic root sections were assessed for atherosclerotic plaque size after staining with Gabe's Aldehyde Fuchsin. Adjacent sections were used to assess plaque cellular content by immunofluorescence staining of macrophages and CD3^+^ T cells by mAb staining for Mac2 (rat anti-mouse, Cedarlane, Canada), and CD3 (MCA1477, AbD Serotec, Germany), respectively. Briefly, slides were blocked with 1% bovine serum albumin (Sigma Aldrich), incubated with primary antibody overnight at 4°C, and secondary detection using the relevant Alexafluor 488-conjugated antibody (Molecular Probes, Life Technologies, Germany). Sections were coverslipped using DAPI-containing Vectashield mounting medium (Vector laboratories, Burlingame, USA). The extent of atherosclerosis throughout the thoracoabdominal aorta was assessed by staining for lipid depositions with Oil-red-O. Briefly, the thoracoabdominal aorta was opened longitudinally, stripped of adventitia and the percentage of lipid deposition was calculated by dividing the stained area by the total thoracoabdominal aortic surface. All images were recorded with a Leica DM 4000B fluorescence microscope and JVC KY-F75U camera. Plaque size, collagen content and cell content were quantified by computerized image analysis (Diskus Software, Hilgers, Germany).

### Serum cholesterol and triglyceride measurement

Serum was analysed for total cholesterol (Amplex Red Cholesterol Assay Kit, Invitrogen, Life Technologies) and triglycerides (EnzyChrom Triglyceride Assay Kit, BioAssay Systems, USA), according to the manufacturer's instructions.

### Statistical analysis

Data are displayed as mean ± SEM. Gaussian distribution of values was checked using D'Agostino and Pearson Normality test. When normality test was passed for values from both experimental groups, an unpaired t test was performed. When normality test failed, a non-parametric Mann-Whitney U test was performed. Results from Treg suppression assays were analyzed by 1-way Analysis of Variance followed by Bonferroni post-hoc test. Results with p<0.05 were considered significant.

## Results

### PD-1 deficiency increases CD4^+^ T cell activation in blood and secondary lymphoid organs

**Figure 1:**
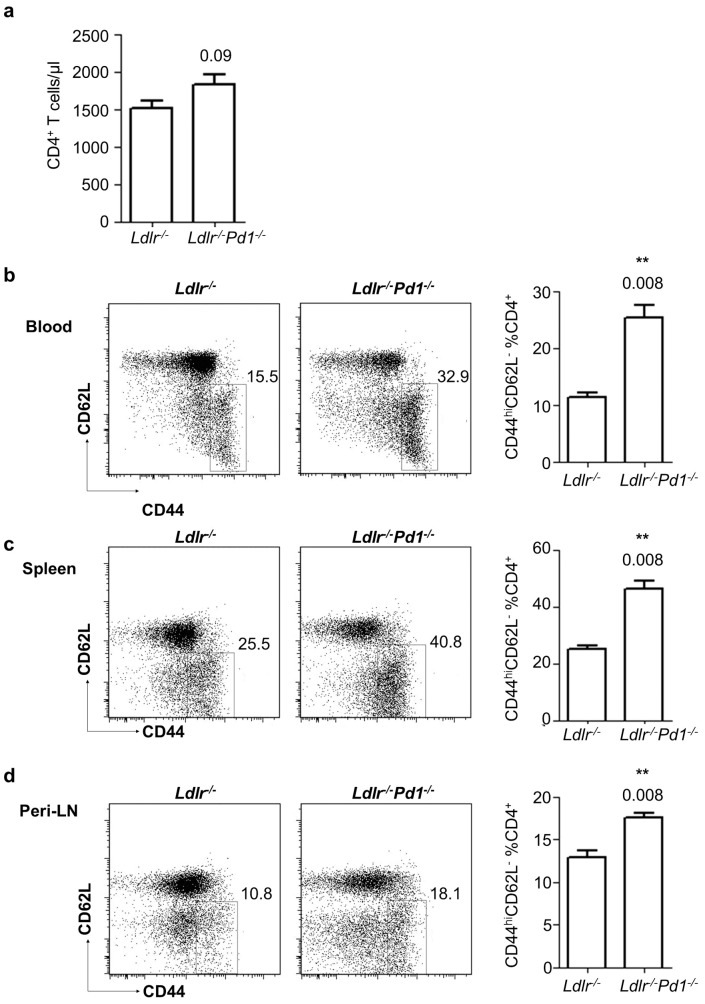
PD-1 deficiency increases CD4^+^ T cell activation in hypercholesterolemic mice. a) Circulating numbers of CD4^+^ T cells in *Ldlr^−^* ^/*−*^ or *Ldlr^−^* ^/*−*^ *Pd1^−^* ^/*−*^ mice after 9 weeks of high fat diet feeding. b-d) Representative FACS dot plots showing CD44 and CD62L expression in pre-gated CD45^+^CD3^+^TCRβ^+^ CD4^+^ T cells and quantitation of frequencies of CD44^hi^CD62L^−^ effector/memory cells among CD4^+^ T cells in blood (b), spleen (c) and peripheral LN (d) after 9 weeks of high fat diet feeding (n = 5/group, representative of 2 independent experiments). **p<0.01.

**Figure 2:**
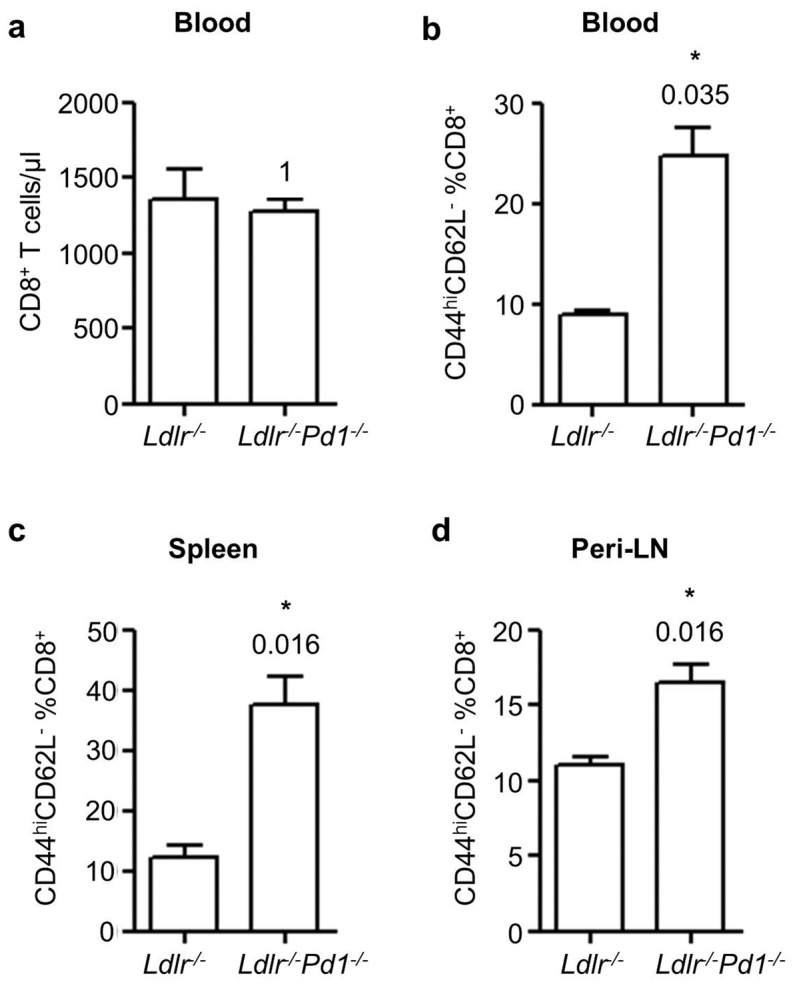
PD-1 deficiency increases CD8^+^ T cell activation in hypercholesterolemic mice. a) Circulating numbers of CD8^+^ T cells in *Ldlr^−^* ^/*−*^ or *Ldlr^−^* ^/*−*^ *Pd1^−^* ^/*−*^ mice after 9 weeks of high fat diet feeding. Quantification of frequencies of CD44^hi^CD62L^−^ effector/memory cells among CD45^+^CD3^+^TCRβ^+^ CD8^+^ T cells in blood (b), spleen (c) and peripheral LN (d) after 9 weeks of high fat diet feeding (n = 5/group). *p<0.05.

To investigate the role of PD-1 in T cell activation and Treg homeostasis during hypercholesterolemia-induced atherosclerosis, *Ldlr^−/−^Pd1^−/−^* deficient mice and control *Ldlr^−/−^* mice were fed a high fat diet for 9 weeks. At the end of this diet period, activation of T cells was evaluated by flow cytometry staining for CD44 and CD62L in circulating blood and single cell suspensions obtained from spleen and peripheral (axillary and inguinal) lymph nodes (LN). Although absolute numbers of circulating CD4^+^ T cells only showed a trend towards increased levels (Figure 1a, p = 0.09), we observed a 2.3-fold increase in the proportion of activated CD44^hi^CD62L^−^ effector/memory cells among CD4^+^ T cells in blood of *Ldlr^−/−^Pd1^−/−^* mice (Figure 1b). Likewise, frequencies of CD44^hi^CD62L^−^ cells among CD4^+^ T cells were elevated by 1.9-fold and 1.4-fold in the spleen and peripheral LN, respectively (Figure 1c, d). Similarly, while circulating levels of CD8^+^ T cell were unaltered between groups (Figure 2a), frequencies of CD44^hi^CD62L^−^ effector/memory T cells were strongly increased in blood, spleen and peripheral LN in *Ldlr^−/−^Pd1^−/−^* mice compared to *Ldlr^−/−^* controls (Figure 2b-d). These data indicate that deficiency in PD-1 leads to widespread CD4^+^ and CD8^+^ T cell activation in hypercholesterolemic mice.

### PD-1 deficiency only moderately affects Th1/Th17 polarization but leads to systemic Treg expansion

**Figure 3:**
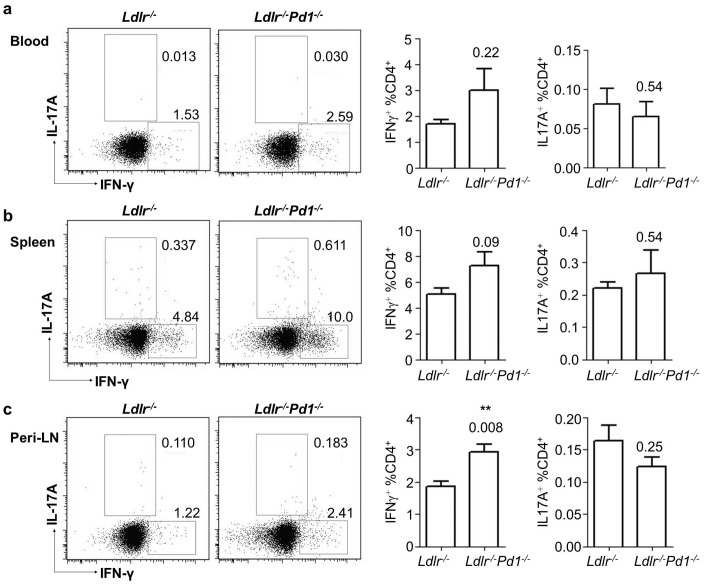
PD-1 deficiency only slightly affects CD4^+^ T cell polarization towards the Th1 lineage. a) Representative FACS dot plots and quantification of frequencies of IL-17A and IFNγ-expressing cells in pre-gated CD45^+^CD3^+^TCRβ^+^ CD4^+^ T cells in blood (a), spleen (b) and peripheral LN (c) of *Ldlr^−^* ^/*−*^ or *Ldlr^−^* ^/*−*^ *Pd1^−^* ^/*−*^ mice after 9 weeks of high fat diet feeding (n = 5/group, representative of 2 independent experiments). **p<0.01.

Both Th1 cells as well as Th17 cells have been shown to modulate the development of atherosclerosis [2], [4]. We therefore evaluated whether the activation of CD4^+^ T cells was associated with a polarization towards the Th1 or Th17 lineage by staining for intracellular IFNγ (Th1) and IL17A (Th17) cytokine expression, not investigated previously. Frequencies of IFNγ-producing CD4^+^ T cells were unaffected but tended to be elevated in blood and spleens (Figure 3a, b), and showed a significant increase in peripheral LN of *Ldlr^−/−^Pd1^−/−^* mice (Figure 3c). Levels of IL17A-producing CD4^+^ T cells were unaffected by PD-1 deficiency in all organs tested (Figure 3a-c). These results unprecedentedly indicate that PD-1 deficiency only moderately affects systemic Th1 but not Th17-differentiation, but is associated with a shift towards pro-atherogenic Th1 cells in peripheral LN.

**Figure 4:**
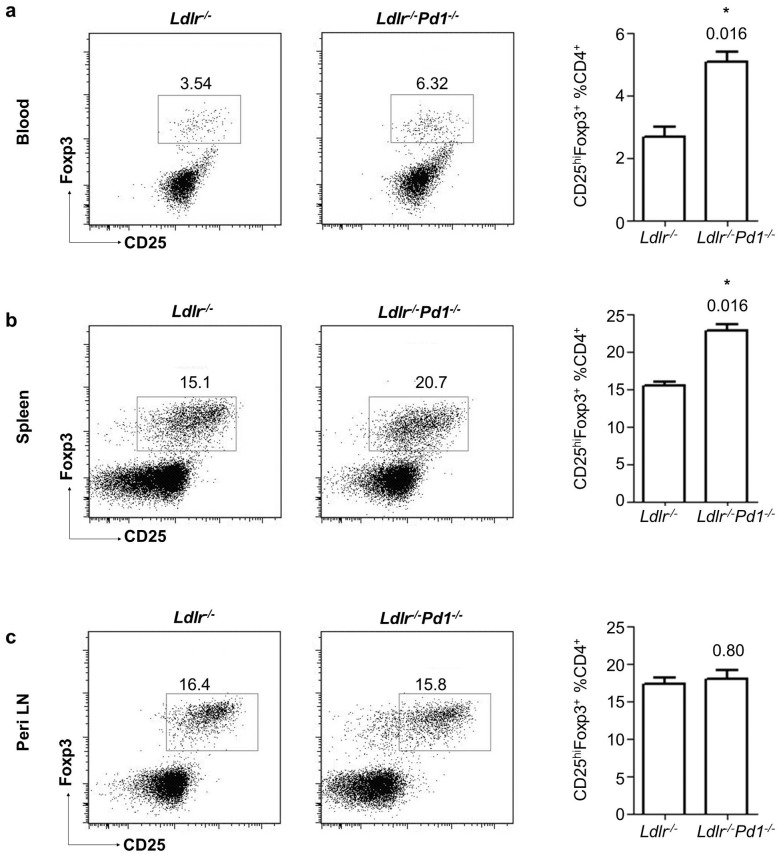
Treg expansion in *Ldlr^−/−^Pd1^−/−^* mice. Representative FACS dot plots and quantification of frequencies of Foxp3 and CD25-expressing cells in pre-gated CD45^+^CD3^+^TCRβ^+^ CD4^+^ T cells in blood (a), spleen (b) and peripheral LN (c) of *Ldlr* ^−/−^ or *Ldlr* ^−/−^ *Pd1* ^−/−^ mice after 9 weeks of high fat diet feeding (n = 5/group, representative of 2 independent experiments). *p<0.05.

Interestingly, also frequencies of atheroprotective CD25^hi^Foxp3^+^ Tregs [8], [10] among CD4^+^ T cells were strongly increased in circulating blood (Figure 4a) and spleens of *Ldlr^−/−^Pd1^−/−^ versus Ldlr^−/−^* mice (Figure 4b), while their proportions were similar in peripheral LN (Figure 4c). These data for the first time indicate that PD-1 deficiency entails a systemic increase in atheroprotective Tregs.

### PD-1 deficiency increases lesional activated T cell and Treg content

**Figure 5:**
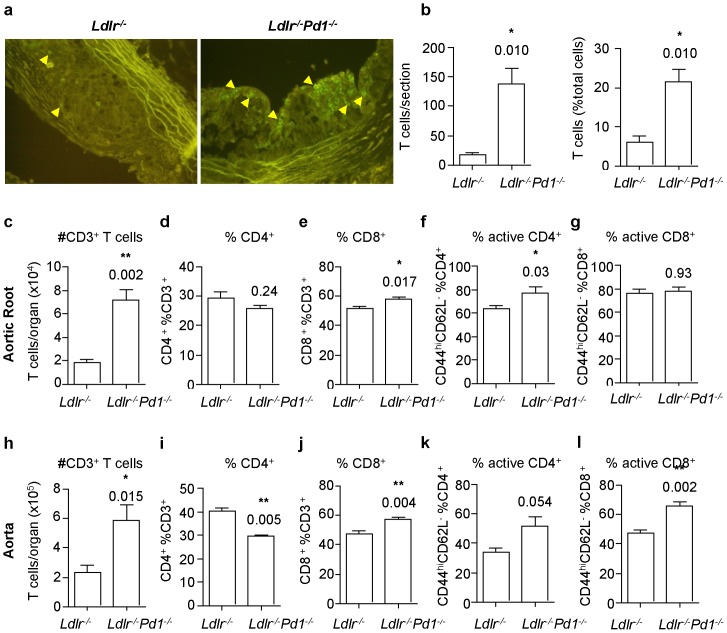
Increased T cell infiltration and activation in atherosclerotic lesions in *Ldlr^−/−^Pd1^−/−^* mice. a) Representative images of anti-CD3 immunofluorescence staining (positive cells are indicated by arrowheads) and b) quantitative analysis of CD3^+^ T cell numbers and frequencies among total plaque cells in atherosclerotic plaques in the aortic sinus of *Ldlr^−/−^* and *Ldlr^−/−^Pd1^−/−^* mice after 9 weeks of high fat diet feeding. c-l) FACS analysis of total CD3^+^ T cell infiltration, CD4^+^ and CD8^+^ expressing cell proportions among total CD3^+^ T cells, and proportion of CD44^hi^CD62L*^−^* activated cells among CD4^+^ and CD8^+^ T cells in single cell suspensions obtained from the aortic root (c-g) or the aorta (h-l) of *Ldlr^−/−^* and *Ldlr^−/−^Pd1^−/−^* mice after 9 weeks of high fat diet feeding. *p<0.05 **p<0.01.

Although systemic immune processes have a role in atherogenesis, local inflammatory T cell responses in atherosclerotic vessels are of paramount importance in lesion formation and progression. We thus evaluated local immune cell accumulation by immunostaining for T cells in cryosections through the aortic sinus. Infiltration of CD3^+^ T cells in atherosclerotic plaques showed a striking 8.2-fold increase in plaques from *Ldlr* ^−*/*−^ *Pd1* ^−*/*−^ mice (Figure 5a, b), where T cells constituted over 20% of total plaques cells, while they represented less than 10% in lesions of *Ldlr* ^−*/*−^ mice (Figure 5b). In order to further obtain a more precise view of T cell responses within lesions, aortic roots and aortae from *Ldlr* ^−*/*−^ and *Ldlr* ^−*/*−^ *Pd1* ^−*/*−^ mice were enzymatically digested into single cell suspensions and analyzed by flow cytometry. In line with immunohistochemistry results, absolute numbers of CD3^+^ T cells were markedly increased in the aortic root (Figure 5c) and the aorta (Figure 5h) in *Ldlr* ^−*/*−^ *Pd1* ^−*/*−^ versus *Ldlr* ^−*/*−^ mice. A slight shift from CD4^+^ to CD8^+^ T cells was noted among total CD3^+^ T cells in aortic roots (Figure 5d, e) and aortae (Figure 5i, j) in *Ldlr* ^−*/*−^ *Pd1* ^−*/*−^ mice. Importantly, a significant increase in the activation of CD4^+^ T cells was found in the aortic root (Figure 5f, p = 0.03) and a strong trend towards increased activation was similarly observed in the aorta (Figure 5k, p = 0.054). Frequencies of activated CD8^+^ T cells were significantly higher in *Ldlr* ^−*/*−^ *Pd1* ^−*/*−^ aortae (Figure 5l, p = 0.002) but unaltered in the aortic root (Figure 5g).

**Figure 6:**
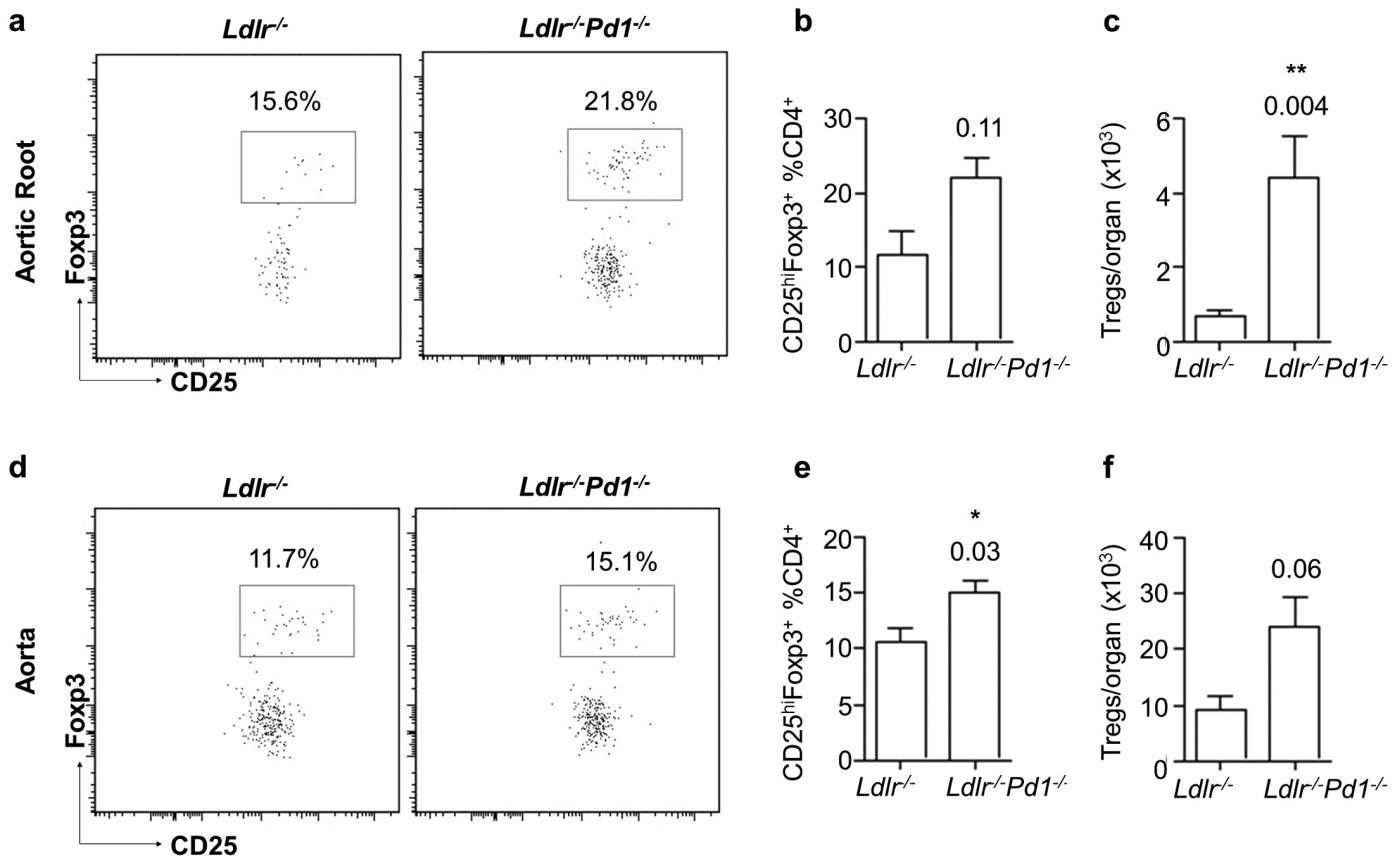
Increased Treg infiltration in atherosclerotic lesions in *Ldlr^−/−^Pd1^−/−^* mice. Representative FACS dot plots (left), proportion of CD25^hi^Foxp3^+^ Tregs among CD4^+^ T cells (middle) and absolute number of CD25^hi^Foxp3^+^ Tregs (right) in the aortic root (a-c) and the aorta (d-f) of *Ldlr^−/−^* and *Ldlr^−/−^Pd1^−/−^* mice after 9 weeks of high fat diet feeding. *p<0.05 **p<0.01.

Notably, also Tregs accumulated to a larger extent in the aortic root and the aorta of *Ldlr^−/−^Pd1^−/−^* compared to *Ldlr^−/−^* mice. Absolute counts of Tregs showed a drastic 6.8-fold increase in the aortic root (Figure 6c, p = 0.004), and a strong albeit non-significant increase in the aorta (Figure 6f, p = 0.06). Similarly, relative Treg frequencies among CD4^+^ T cells were increased in the aorta, and showed a clear trend towards increased proportions in the aortic root. These data provide first evidence that PD-1 deficiency leads to an accumulation of activated CD4^+^ and CD8^+^ T cells also in atherosclerotic vessels, paralleled by an expansion of Tregs.

### PD-1 deficient Tregs efficiently suppress T cell proliferation *in vitro*

**Figure 7:**
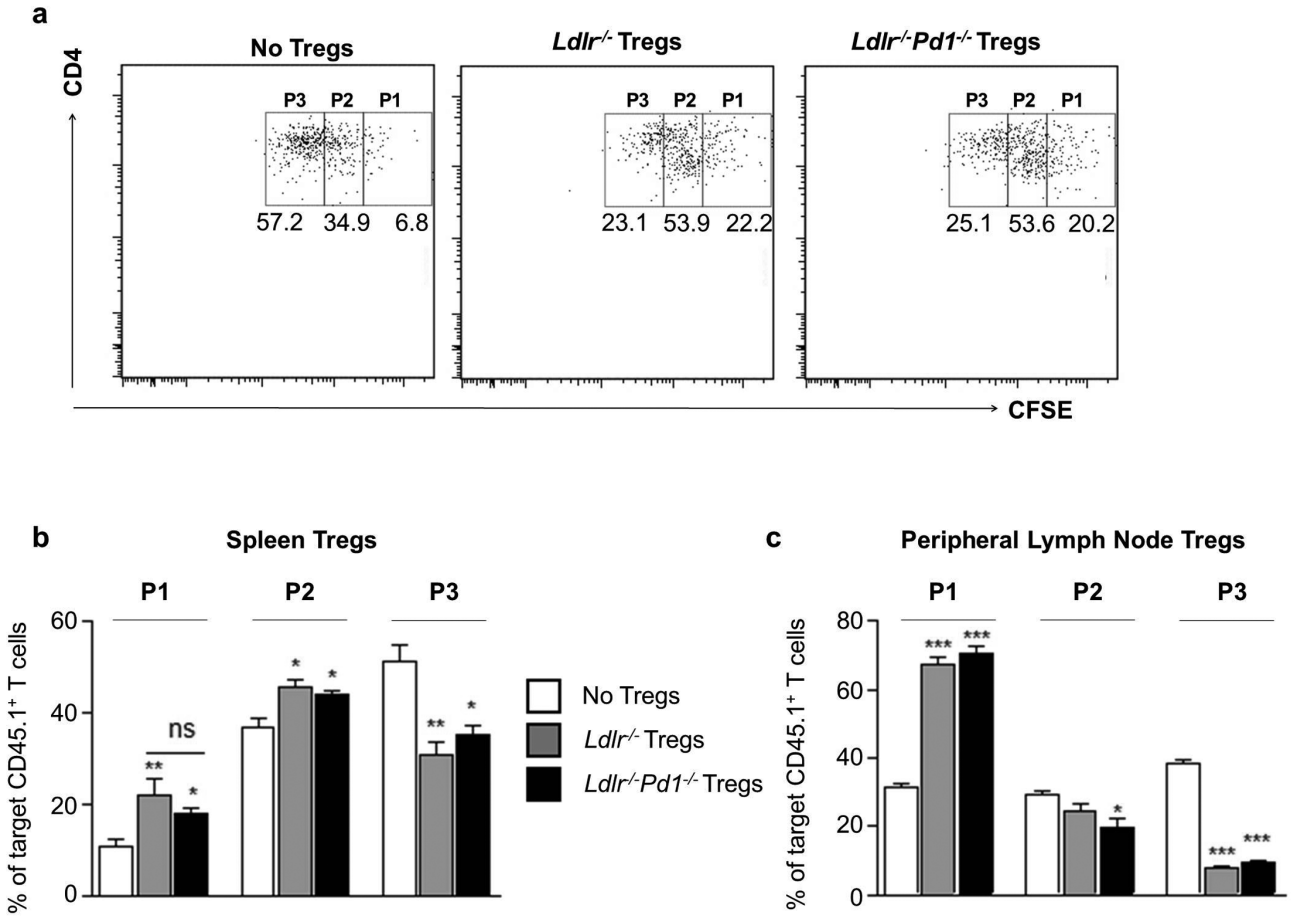
PD-1-deficient Tregs display normal suppressive function *in vitro.* Target naïve CD4^+^ T cells were mixed with *Ldlr^−^* ^/*−*^ or *Ldlr^−^* ^/*−*^ *Pd1^−^* ^/*−*^ Tregs from spleen or peripheral lymph nodes at a ratio of 2∶1. Representative FACS dot plots showing CD4 expression and CFSE fluorescence in pre-gated target CD45.1^+^ T cells; gates P1 to P3 depict CFSE dye dilution following T cell proliferation (a). Quantitative analysis of frequencies of target T cells in each gate after incubation with Tregs from spleen (b) or peripheral LN (c). *p<0.05 **p<0.01 ***p<0.001 *vs* no Tregs. Tregs from n = 3 mice per group were assessed in triplicates.

To further investigate the functionality of PD-1-deficient Tregs, *in vitro* suppression assays were performed. *Ldlr^−/−^Pd1^−/−^* Tregs isolated from spleen (Figure 7a, b) or LN (Figure 7c) were equally potent in suppressing anti-CD3/anti-CD28-induced T cell proliferation as Tregs from *Ldlr^−/−^* mice, indicating that PD-1 deficiency does not affect suppressive functions of Tregs.

### PD-1 deficiency increases atherosclerosis and hypercholesterolemia

**Figure 8:**
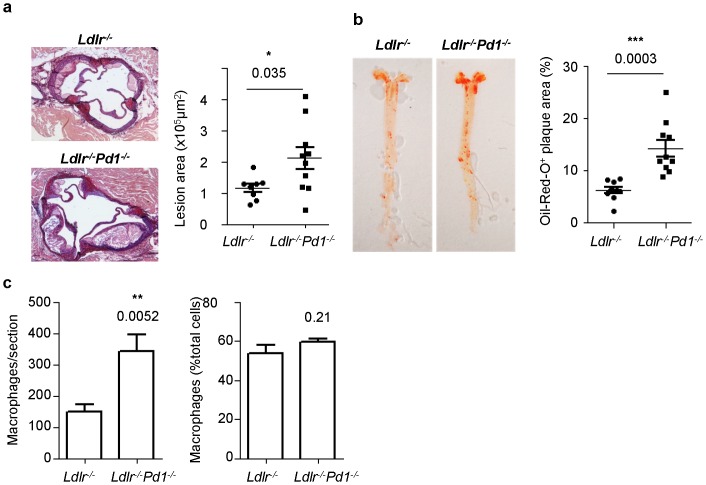
Increased atherosclerotic lesion formation in *Ldlr^−/−^Pd1^−/−^* mice. a) Representative images and quantitative analysis of atherosclerotic lesion size in the aortic sinus of *Ldlr* ^−/−^ or *Ldlr* ^−/−^ *Pd1* ^−/−^ mice after 9 weeks of high fat diet feeding. b) Representative images of Oil-Red-O stained aortae and quantitative analysis of atherosclerotic lesion size in the aorta of *Ldlr* ^−/−^ or *Ldlr* ^−/−^ *Pd1* ^−/−^ mice after 9 weeks of high fat diet feeding (n = 8–10/group). (c) Quantitative analysis of Mac2^+^ monocyte/macrophage numbers and frequencies among total plaque cells in the aortic sinus of *Ldlr^−/−^* and *Ldlr^−/−^Pd1^−/−^* mice after 9 weeks of high fat diet feeding (n = 5–9/group). *p<0.05 **p<0.01 ***p<0.001.

Finally, we analyzed atherosclerotic lesion formation in *Ldlr^−/−^Pd1^−/−^* mice. After 9 weeks of high fat diet feeding, atherosclerotic plaque size was strongly increased in *Ldlr^−/−^Pd1^−/−^* compared to *Ldlr^−/−^* mice both in the aortic sinus (Figure 8a) and the aorta (Figure 8b). Although absolute macrophage numbers per section were increased in plaques from *Ldlr^−/−^Pd1^−/−^* mice, in line with an increased lesion size, the relative proportion of Mac2^+^ macrophages among total plaque cells was similar between *Ldlr^−/−^* and *Ldlr^−/−^Pd1^−/−^* mice (Figure 8c). Notably, cholesterol levels were noted to be significantly increased in serum of *Ldlr^−/−^Pd1^−/−^* mice, while triglyceride levels showed a non-significant trend towards raised levels (Table 1). These results indicate that PD-1 deficiency is associated with strongly exacerbated atherosclerotic lesion development, together with aggravated dyslipidemia.

**Table 1:** Lipid levels of *Ldlr^−/−^* or *Ldlr^−/−^Pd1^−/−^* mice after 9 weeks of high fat diet feeding.

	*Ldlr^−^* ^/*−*^ mice	*Ldlr^−^* ^/*−*^ *Pd1^−^* ^/*−*^ mice	p-value
Serum cholesterol (μg/ml)	13475 ± 468	16640 ± 864	<0.01
Serum triglycerides (mmol/L)	6.693 ± 0.137	7.338 ± 0.302	0.09

## Discussion

In the present study, we show that PD-1 deficiency leads to widespread CD4^+^ T cell activation during atherogenesis, but is also associated with a strong expansion of atheroprotective Tregs both systemically and in atherosclerotic vessels not evidenced previously. The net outcome of PD-1 deficiency, however, was a markedly exacerbated atherosclerotic lesion formation in *Ldlr^−/−^* mice. Besides providing confirmation of the critical role of PD-1 signaling in atherosclerotic lesion development, our results highlight the complex role of co-stimulatory and co-inhibitory pathways in controlling the balance of pro-atherogenic *versus* atheroprotective T cell responses during atherogenesis.

Not unexpectedly, T cells showed a widespread activation in atherosclerotic *Ldlr^−/−^Pd1^−/−^ versus Ldlr^−/−^* mice, as revealed by strongly increased frequencies of CD44^hi^CD62L^−^ effector/memory cells among CD4^+^ and CD8^+^ T cells both systemically and at atherosclerosis-prone vascular sites. These findings are in line with previous reports in *Ldlr^−/−^Pd1^−/−^* [10] and *Ldlr^−/−^Pdl1/Pdl2^−/−^* [11] mice, and consistent with the important role of PD-1 signaling in taming T cell activation in inflammatory and autoimmune diseases [12]. Besides activation, polarization of T cell subsets is critical in atherogenesis. In particular, Th1 polarization is thought to drive disease progression both in murine models [2] and humans [13]. CD4^+^ T cell polarization was only moderately shifted towards the IFNγ-producing Th1 effector T cell lineage in specific locations, such as the spleen and peripheral LN, suggesting a somewhat stronger pro-inflammatory and pro-atherogenic profile. This notion is in line with previous reports, showing that PD-1 ligation can inhibit the expression of transcription factors involved in Th1 cell functions, including Eomes and Tbet [12], and the increased Th1 polarization following PD-1 blockade in a murine model of experimental autoimmune encephalomyelitis [14]. Although PD-1 signaling has been proposed as an inhibitory mechanism in Th17 cell differentiation [15], we did not observe any significant modulation of Th17 polarization in atherosclerotic *Ldlr^−/−^Pd1^−/−^*mice. It should be noted, however, that PD-1 signaling can also affect T cell expression of other pro-atherogenic factors, such as TNFα [12], which were not tested in our study.

Notably, levels of CD25^hi^Foxp3^+^ Tregs were strongly increased in *Ldlr^−/−^Pd1^−/−^*mice after 9 weeks of high fat diet, with PD -1 deficient Tregs from various sources showed intact suppressive functions *in vitro*. Previous studies have suggested differing roles of PD-1 signaling in Treg differentiation and function. In patients infected with Hepatitis C virus, increased PD-1 expression on Tregs correlated with lower Treg numbers and clinical markers of immune modulation, which *in vitro* could be overcome by blocking PD-L1/PD-1 interactions [16]. Along these lines, it was demonstrated that PD-1 and PD-L1 negatively regulate the differentiation and suppressive functions of a particular Treg subset, namely follicular Tregs [17]. However, other evidence points towards the opposite role of PD-L1/PD-1 signaling in Treg homeostasis. Particularly, PD-L1 has been shown to promote induced Treg differentiation and function [18]. Discrepancies between these reports could stem from bi-directional signaling in the PD-L1/PD-1 pathway [12]. Besides signaling cascades triggered by PD-1 in T cells, PD-L1, is also able to induce intracellular signaling upon PD-1 binding, and has notably been proposed to increase anti-inflammatory properties of dendritic cells, such as IL-10 secretion [19]. Of note, PD-L1 is also expressed by Tregs [12], and signaling through PD-L1 might also affect Treg expansion and function. Moreover, besides a putative direct role of PD-1 signaling in Treg homeostasis, PD-1 may also modulate Treg expansion through down-modulation of CD28 or IL-2 signaling [12], required for Treg differentiation as well as T cell activation [20], [21]. In line, CD28 signaling is critical for both effector T cell activation and development of Treg responses during atherogenesis [8], and results from several reports suggest that in hypercholesterolemic mice, the extent of Treg expansion is tightly linked to the overall extent of CD4^+^ T cell activation [7]–[9].

The outcome of PD-1 deficiency in *Ldlr^−/−^* mice was a net increase in vascular inflammation and atherosclerosis, suggesting that overwhelming CD4^+^ and CD8^+^ T cell activation could not be compensated by Treg-mediated immune modulation in hypercholesterolemic *Ldlr^−/−^Pd1^−/−^* mice. This observation might potentially be of importance, as it suggests that the efficiency of therapeutic Treg expansion, which has been investigated in pre-clinical models of atherosclerosis [20], might be hampered in an environment highly favorable of effector T cell activation. Importantly, we observed a striking increase in T cell infiltration of atherosclerotic plaques in *Ldlr^−/−^Pd1^−/−^* mice. This might reflect increased T cell recruitment following peripheral activation, as well as enhanced *in situ* T cell proliferation and/or reduced apoptosis, two processes that are modulated by PD-1 signaling [12]. Previous reports have demonstrated that disruption of PD-L1/PD-1 signaling entailes an increased accumulation of CD8^+^ T cells in atherosclerotic lesions [10], [11]. Our own data show a similar shift from CD4^+^ to CD8^+^ T cells in atherosclerotic vessels of *Ldlr^−/−^Pd1^−/−^* mice, associated with increased CD8^+^ T cell activation in aortae, blood, spleen and lymph nodes of *Ldlr^−/−^Pd1^−/−^* mice. Even though the role of CD8^+^ T cells in atherogenesis has long remained enigmatic, a recent report [21] and our own observations (Zernecke et al., unpublished data) revealed that CD8^+^ T cell depletion in *Apoe^−/−^* or *Ldlr^−/−^* mice reduced atherogenesis. Hence, increased CD8^+^ T cell activation and infiltration into plaques likely contributes to increased atherosclerosis in *Ldlr^−/−^Pd1^−/−^*mice.

Besides increased vascular inflammation, we also observed aggravated dyslipidemia in *Ldlr^−/−^Pd1^−/−^* mice with strongly raised blood cholesterol and a trend towards increased triglyceride levels. Inflammation has emerged as a major contributor to the metabolic syndrome, including dyslipidemia [22], and evidence indicates that the balance of regulatory and pro-inflammatory T cell immunity affects diet-induced hyperlipidemia. For instance, Treg depletion in mice has recently been shown to alter lipoprotein metabolism in the liver and to promote hypercholesterolemia and atherosclerosis [23]. Although we observed an expansion of Tregs in *Ldlr^−/−^Pd1^−/−^* mice, net effects of PD-1 deficiency seemed to clearly favor the pro-inflammatory arm of the regulatory/pro-inflammatory T cell response, therefore one could speculate that increased systemic T cell-dependent inflammation, including sites such as the liver or adipose tissue, might enhance diet-associated dyslipidemia and exacerbate atherogenesis in *Ldlr^−/−^Pd1^−/−^* mice.

In conclusion, our results highlight the dual role of co-stimulatory and co-inhibitory signals in the control of both pro-atherogenic and atheroprotective T cell activation in hypercholesterolemia, and corroborate a critical role of PD-1 signaling in limiting atherogenesis.
